# Dense Quantum Measurement Theory

**DOI:** 10.1038/s41598-019-43250-2

**Published:** 2019-05-01

**Authors:** Laszlo Gyongyosi, Sandor Imre

**Affiliations:** 1 School of Electronics and Computer Science, University of Southampton, Southampton, SO17 1BJ UK; 2 Department of Networked Systems and Services, Budapest University of Technology and Economics, Budapest, H-1117 Hungary; 3 MTA-BME Information Systems Research Group, Hungarian Academy of Sciences, Budapest, H-1051 Hungary

**Keywords:** Quantum mechanics, Computer science, Quantum information, Mathematics and computing, Computational science

## Abstract

Quantum measurement is a fundamental cornerstone of experimental quantum computations. The main issues in current quantum measurement strategies are the high number of measurement rounds to determine a global optimal measurement output and the low success probability of finding a global optimal measurement output. Each measurement round requires preparing the quantum system and applying quantum operations and measurements with high-precision control in the physical layer. These issues result in extremely high-cost measurements with a low probability of success at the end of the measurement rounds. Here, we define a novel measurement for quantum computations called dense quantum measurement. The dense measurement strategy aims at fixing the main drawbacks of standard quantum measurements by achieving a significant reduction in the number of necessary measurement rounds and by radically improving the success probabilities of finding global optimal outputs. We provide application scenarios for quantum circuits with arbitrary unitary sequences, and prove that dense measurement theory provides an experimentally implementable solution for gate-model quantum computer architectures.

## Introduction

Quantum measurement is a crucial subject in quantum computation and communication^1–31^. The aim of quantum measurement is to extract valuable and useable information from the measured quantum system. The measurement operator connects the quantum world and our traditional, classical world. While the input of the measurement can be a superposed or entangled quantum system, the output of the measurement is classical information (i.e., bitstrings). Quantum measurements can be performed in different ways, for example via projective^32–39^ or POVM (positive-operator valued measure) measurements^35,40–46^.

Quantum measurement is required element in high-complexity quantum computations, in high-performance quantum information processing and in quantum computer architectures. The main issues of current quantum measurement strategies are the high number of measurement rounds and the probability of successfully finding a global optimal measurement output. The necessity of a high number of measurement rounds requires preparing the input quantum system and applying quantum operations with high-precision control in the physical layer through several rounds, which results in a high-cost procedure overall that is not tractable in any experimental setting. The repetition of a measurement round therefore requires in each round the careful preparation of a quantum register of quantum states that are then fed into a quantum circuit that realizes an arbitrary unitary sequence. In each round, the output of the quantum circuit is measured by a measurement array *M*, which produces a classical output string *z*. The aim is then to find a global optimal output z* that describes the properties of the output quantum system with the highest accuracy according to quality measurement functions. An example of a high-cost application of standard measurement is measuring the output of a quantum circuit applied to realize quantum computations where the quantum circuit is set to perform a unitary operation *U*. Without loss of generality, the *n*-length input quantum system $|X\rangle$ of the quantum circuit is assumed to be a superposed quantum system that is fed into the circuit. Then, the *n*-length output quantum system $|Y\rangle =U|X\rangle$ is measured by the measurement operator *M*, which produces a string *z* and, after repeating the procedure *R*_0_ times, yields the global optimal string z* with success probability ${{\rm{\Pr }}}_{{R}_{0}}({z}^{\ast })$. Assuming that *U* is an arbitrary quantum circuit and *M* is a standard measurement, the measurement procedure requires high repetition numbers, while the success probability remains low (An example is the application of standard quantum measurements in quantum computers, where for ${R}_{0}\approx 100$ standard measurement rounds, the achievable success probability is approximately ${{\rm{\Pr }}}_{{R}_{0}}({z}^{\ast })\ge 0.01$^13^). Since each measurement round requires high-cost and high-precision quantum state preparations and quantum operations, the total cost to find the global optimal z* is very high in a practical setting. To avoid the issues of a high number of measurement rounds and the low success probability of quantum measurements, a novel measurement is essential for quantum computations.

Here, we define a novel measurement for quantum computations called dense quantum measurement. The dense measurement strategy aims at fixing the drawbacks of standard quantum measurements by achieving a radical reduction in the number of necessary measurement rounds and by significantly improving the success probabilities of finding global optimal outputs (see Theorem 1 for the system model). Dense quantum measurement requires only $R\ll {R}_{0}$ measurement rounds, such that *R* rounds leads to a success probability of ${\rm{\Pr }}({z}^{\ast })\gg {{\rm{\Pr }}}_{{R}_{0}}({z}^{\ast })$. The dense measurement strategy is rooted in the theory of compressed sensing^47–50^, which allows recovering noisy signals with a high efficiency in the field of traditional communications. Dense quantum measurement utilizes an *M*_*r*_ randomized measurement operator that is defined as an *n*-bit length vector ${M}_{r}={({b}_{1}{M}_{B},\ldots ,{b}_{n}{M}_{B})}^{T}$, where *b*_*i*_ is a random variable, ${b}_{i}\in \{0,1\}$, ${\rm{\Pr }}(0)={\rm{\Pr }}(1)=0.5$, associated with the measurement of the *i*-th quantum state of the output quantum system, while *M*_*B*_ is a quantum measurement in the computational basis *B*; thus, ${b}_{i}{M}_{B}=0$ if ${b}_{i}=0$ and ${b}_{i}{M}_{B}={M}_{B}$ if ${b}_{i}=1$. As follows, the *M*_*B*_ measurement in the computational basis is discarded if ${b}_{i}=0$. Then, the measurement result is post-processed via unit ${\mathscr{P}}$ that integrates algorithms to determine the global optimal string z* from the results of the randomized measurements.

As we prove (see Theorem 2), the number *R*_0_ of standard measurement rounds can be reduced to $R=\alpha {Z}^{2}K\,{\mathrm{log}}^{4}(n)$ dense measurement rounds for an arbitrary quantum circuit, where $K\ge {L}_{0}(S)$ and $K\ll n$, while $\alpha  > 0$, $Z > 0$ are constants. At this number of measurement rounds, the success probability is ${\rm{\Pr }}({z}^{\ast })=1-{n}^{-{\mathrm{log}}^{3}(n)}\approx 1$ for any practical value of *n*. We also prove that if the output of the quantum circuit is a computational basis quantum state, then *R*_0_ can be reduced to $R={\mathscr{O}}(\gamma K\,\mathrm{log}(\frac{10n}{K}))$ dense measurement rounds, where $\gamma  > 0$ is a constant, such that ${\rm{\Pr }}({z}^{\ast })=1-2\,\exp (\,-\,R)\approx 1,$ for any *R* (see Theorem 3).

The novel contributions of our manuscript are as follows:

1. We define a novel quantum measurement theory called dense quantum measurement.
2. We prove that dense measurement reduces the number of required measurement rounds to find a global optimal output.
3. We prove that dense measurement significantly improves the success probability of finding a global optimal output.
4. We provide an application scenario for quantum circuits with arbitrary unitary sequences, and for the dense measurement of computational basis quantum states in gate-model quantum computer environment.
5. We reveal that the primary advantages of dense quantum measurement theory are the significantly lower measurement rounds and significantly higher success probabilities.

This manuscript is organized as follows. In Section 2, the related works are summarized. In Section 3, the problem statement is given. In Section 4, preliminaries are summarized. Section 5 proposes the theorems and proofs. Section 6 provides a performance evaluation. Finally, Section 7 concludes the paper. Supplemental information is included in the Appendix.

## Related Works

The related works on quantum measurement theory, gate-model quantum computers and compressed sensing are summarized as follows.

### Quantum Measurement Theory

Quantum measurement has a fundamental role in quantum mechanics with several different theoretical interpretations^32–44^. The measurement of a quantum system collapses of the quantum system into an eigenstate of the operator corresponding to the measurement. The measurement of a quantum system produces a measurement result, the expected values of measurement are associated with a particular probability distribution.

In quantum mechanics several different measurement techniques exist. In a projective measurement^32–39^, the measurement of the quantum system is mathematically interpreted by projectors that project any initial quantum state onto one of the basis states. The projective measurement is also known as von Neumann measurement^32^. In our manuscript the projective measurement with no post-processing on the measurement results is referred to as *standard measurement* (It is motivated by the fact, that in a gate-model quantum computer environment the output quantum system is measured with respect to a particular computational basis).

The von Neumann measurements are a special case of a more general measurement, the POVM measurement^35,40–44^. Without loss of generality, the POVM is a generalized measurement that can be interpreted as a von Neumann measurement that utilizes an additional quantum system (called ancilla). The POVM measurement is mathematically described by a set of positive operators such that their sum is the identity operator^51–53^. The POVM measurements therefore can be expressed in terms of projective measurements (see also Neumark’s dilation theorem^54–56^).

Another subject connected to quantum measurement theory is quantum-state discrimination^57–61^ that covers the distinguishability of quantum states, and the problem of differentiation between non-orthogonal quantum states.

### Gate-Model Quantum Computers

The theoretical background of the gate-model quantum computer environment utilized in our manuscript can be found in^12^ and^13^.

In^13^, the authors studied the subject of objective function evaluation of computational problems fed into a gate-model quantum computer environment. The work focuses on a qubit architectures with a fixed hardware structure in the physical layout. In the system model of a gate-model quantum computer, the quantum computer is modeled as a sequence of unitary operators (quantum gates). The quantum gates are associated with a particular control parameter called the gate parameter. The quantum gates can process one-qubit length and multi-qubit length quantum systems. The input quantum system (particularly a superposed quantum system) of the quantum circuit is transformed via a sequence of unitaries controlled via the gate parameters, and the output qubits are measured by a measurement array. The measurement in the model is realized by a projective measurement applied on a qubits that outputs a logical bit with value zero or one for each measured qubit. The result of the measurement is therefore a classical bitstring. The output bitstring is processed further to estimate the objective function of the quantum computer. The work also induces and opens several important optimization questions, such as the optimization of quantum circuits of gate-model quantum computers, optimization of objective function estimation, measurement optimization and optimization of post-processing in a gate-model quantum computer environment. In our particular work we are focusing on the optimization of the measurement phase.

An optimization algorithm related to gate-model quantum computer architectures is defined in^12^. The optimization algorithm is called “Quantum Approximate Optimization Algorithm” (QAOA). The aim of the algorithm is to output approximate solutions for combinatorial optimization problems fed into the quantum computer. The algorithm is implementable via gate-model quantum computers such that the depth of the quantum circuit grows linearly with a particular control parameter. The work also proposed the performance of the algorithm at the utilization of different gate parameter values for the unitaries of the gate-model computer environment.

In^62^, the authors studied some attributes of the QAOA algorithm. The authors showed that the output distribution provided by QAOA cannot be efficiently simulated on any classical device. A comparison with the “Quantum Adiabatic Algorithm” (QADI)^63,64^ is also proposed in the work. The work concluded that the QAOA can be implemented on near-term gate-model quantum computers for optimization problems.

An application of the QAOA algorithm to a bounded occurrence constraint problem “Max E3LIN2” can be found in^15^. In the analyzed problem, the input is a set of linear equations each of which has three boolean variables, and each equation outputs whether the sum of the variables is 0 or is 1 in a mod 2 representation. The work is aimed to demonstrate the capabilities of the QAOA algorithm in a gate-model quantum computer environment.

In^65^, the authors studied the objective function value distributions of the QAOA algorithm. The work concluded, at some particular setting and conditions the objective function values could become concentrated. A conclusion of the work, the number of running sequences of the quantum computer can be reduced.

In^66^, the authors analyzed the experimental implementation of the QAOA algorithm on near-term gate-model quantum devices. The work also defined an optimization method for the QAOA, and studied the performance of QAOA. As the authors found, the QAOA can learn via optimization to utilize non-adiabatic mechanisms.

In^67^, the authors studied the implementation of QAOA with parallelizable gates. The work introduced a scheme to parallelize the QAOA for arbitrary all-to-all connected problem graphs in a layout of qubits. The proposed method was defined by single qubit operations and the interactions were set by pair-wise CNOT gates among nearest neighbors. As the work concluded, this structure allows for a parallelizable implementation in quantum devices with a square lattice geometry.

In^14^, the authors defined a gate-model quantum neural network. The gate-model quantum neural network describes a quantum neural network implemented on gate-model quantum computer. The work focuses on the architectural attributes of a gate-model quantum neural network, and studies the training methods. A particular problem studied in the work is the classification of classical data sets which consist of bitstrings with binary labels. In the architectural model of a gate-model quantum neural network, the weights are represented by the gate parameters of the unitaries of the network, and the training method acts these gate parameters. As the authors stated, the gate-model quantum neural networks represent a practically implementable solution for the realization of quantum neural networks on near-term gate-model quantum computer architectures.

In^68^, the authors defined a quantum algorithm that is realized via a quantum Markov process. The analyzed process of the work was a quantum version of a classical probabilistic algorithm for *k*-SAT defined in^69^. The work also studied the performance of the proposed quantum algorithm and compared it with the classical algorithm.

For a review on the noisy intermediate-scale quantum (NISQ) era and its technological effects and impacts on quantum computing, see^1^.

The subject of quantum computational supremacy (tasks and problems that quantum computers can solve but are beyond the capability of any classical computer) and its practical implications are studied in^2^. For a work on the complexity-theoretic foundations of quantum supremacy, see^3^.

A comprehensive survey on quantum channels can be found in^23^, while for a survey on quantum computing technology, see^70^.

### Compressed Sensing

In traditional information processing, compressed sensing^47^ is a technique to reduce the sampling rate to recover a signal from fewer samples than it is stated by the Shannon-Nyquist sampling theorem (that states that the sampling rate of a continuous-time signal must be twice its highest frequency for the reconstruction)^47–50^. In the framework of compressed sensing, the signal reconstruction process exploits the sparsity of signals (in the context of compressed sensing, a signal is called sparse if most of its components are zero)^50,71–75^. Along with the sparsity, the restricted isometry property^50,71,75^ is also an important concept of compressed sensing, since, without loss of generality, this property makes it possible to yield unique outputs from the measurements of the sparse inputs. The restricted isometry property is also a well-studied problem in the field of compressed sensing^76–80^.

A special technique within compressed sensing is the so-called “1-bit” compressed sensing^81–83^, where 1-bit measurements are applied that preserve only the sign information of the measurements.

The application of compressed sensing covers the fields of traditional signal processing, image processing and several different fields of computational mathematics^84–91^.

The dense quantum measurement theory proposed in our manuscript also utilizes the fundamental concepts of compressed sensing. However, in our framework the primary aims are the reduction of the measurement rounds required to determine a global optimal output at arbitrary unitaries, and the boosting of the success probability of finding a global optimal output at a particular measurement round. The results are illustrated through a gate-model quantum computer environment.

## Problem Statement

Let $|X\rangle$ be the superposed input system of a quantum circuit with a *QG* quantum gate structure, formulated by *n* quantum states, as

1 $$|X\rangle =\frac{1}{\sqrt{{d}^{n}}}\,\sum _{z}\,|z\rangle ,$$

where *d* is the dimension of the quantum system, $|z\rangle$ is a computational basis state and $U(\overrightarrow{\theta })$ is the unitary operation of *QG*, defined as a sequence of *L* unitaries

2 $$U(\overrightarrow{\theta })={U}_{L}\,({\theta }_{L})\,{U}_{L-1}\,({\theta }_{L-1}),\ldots ,{U}_{1}\,({\theta }_{1}),$$

where $\overrightarrow{\theta }$ is the *L*-dimensional vector of the gate parameters of the unitaries (gate parameter vector):

3 $$\overrightarrow{\theta }={({\theta }_{1},\ldots ,{\theta }_{L})}^{T}.$$

In (2), an *i*-th unitary gate ${U}_{i}({\theta }_{i})$ is evaluated as

4 $${U}_{i}\,({\theta }_{i})=\exp \,(\,-\,i{\theta }_{i}P),$$

where *P* is a generalized Pauli operator formulated by the tensor product of Pauli operators $\{{\sigma }_{X},{\sigma }_{Y},{\sigma }_{Z}\}$.

In a standard measurement setting, the $|Y\rangle$ output of *QG* is

5 $$|Y\rangle =U\,(\overrightarrow{\theta })\,|X\rangle $$

measured by a *M* measurement operator, which yields an output string *z* as

6 $$z=M\,|Y\rangle .$$

The global optimal output string z* is an output string that yields the optimal estimation $C({z}^{\ast })$ at a particular objective function *C* fed into the quantum circuit as a maximization problem

7 $$C\,({z}^{\ast })=\mathop{{\rm{\max }}}\limits_{\forall m}\,C\,({z}_{m}),$$

where $C({z}_{m})$ is the estimate yielded in an *m*-th measurement round, $m=1,\ldots ,{R}_{0}$, while *z*_*m*_ is the output string yielded in the *m*-th round.

Without loss of generality, after *R*_0_ measurement rounds, the probability that the global optimal output string z* is determined is ${{\rm{\Pr }}}_{{R}_{0}}({z}^{\ast })$; thus, $C({z}^{\ast })$ can be found with the same success probability,

8 $${{\rm{\Pr }}}_{{R}_{0}}C\,({z}^{\ast })={{\rm{\Pr }}}_{{R}_{0}}\,({z}^{\ast }).$$

The problems connected to the general measurement strategy to find z* are the high number of *R*_0_ repetitions and the low ${\Pr }_{{R}_{0}}({z}^{\ast })$ success probability. Consequently, the standard measurement procedure requires high-cost quantum state preparations, the application of high-cost measurement arrays and high-precision control and calibrations in the physical layer.

Problems 1–3 summarize the problems to be solved.

### **Problem 1**

(*System Model*). *Define a novel quantum measurement strategy for the significant reduction of the R*_0_ *measurement rounds of standard measurements and for the significant improvement of the* ${{\rm{\Pr }}}_{{R}_{0}}({z}^{\ast })$ success probability in determining a global optimal output z*.

### **Problem 2**

(*General application*). *Define R and* ${\rm{\Pr }}({z}^{\ast })$ *for an arbitrary quantum circuit with* $U(\overrightarrow{\theta })$. *Prove the number R of measurement rounds*, $R\ll {R}_{0}$, *and the* ${\rm{\Pr }}({z}^{\ast })$ *success probability*, ${\rm{\Pr }}({z}^{\ast })\gg {{\rm{\Pr }}}_{{R}_{0}}({z}^{\ast })$.

### **Problem 3**

(*Dense measurement of computational basis quantum states*). *Define R and* ${\rm{\Pr }}({z}^{\ast })$ *for an arbitrary quantum circuit with* $U(\overrightarrow{\theta })={U}_{B}$, *where U*_*B*_ *sets the computational basis B* (*Throughout the manuscript*, *the term “computational basis” refers to a basis B*, *for which* ${L}_{0}(S)\le K$ *holds at a given* $S=BX$, *where X is an input system*). *Prove the number R of measurement rounds*, $R\ll {R}_{0}$, *and the* ${\rm{\Pr }}({z}^{\ast })$ *success probability*, ${\rm{\Pr }}({z}^{\ast })\gg {{\rm{\Pr }}}_{{R}_{0}}({z}^{\ast })$.

The resolutions of Problems 1–3 are given in Theorems 1–3, respectively.

## Preliminaries

### Sub-Gaussian Distributions

A random variable *X* is sub-Gaussian, if for the probability distribution of *X*,

9 $${\rm{\Pr }}\,(|X|\ge \kappa )\le {C}_{1}{e}^{-{C}_{2}{\kappa }^{2}}$$

holds for $\forall \kappa  > 0$, where

10 $${C}_{1},{C}_{2} > 0$$

are sub-Gaussian parameters.

By theory, if *X* is sub-Gaussian with

11 $${\mathbb{E}}\,(X)=0,$$

then there exists a constant c* depending on only *C*_1_, *C*_2_ such that

12 $${\mathbb{E}}\,(\exp (\eta X)\le \exp \,({c}^{\ast }{\eta }^{2}))$$

for $$\forall \eta \in {\rm{R}}$$.

If (12) holds, then (11) is satisfied such that the *C*_1_ sub-Gaussian parameter of *X* is

13 $${C}_{1}=2,$$

and *C*_2_ is as

14 $${C}_{2}=\frac{1}{4{c}^{\ast }}.$$

An $M\times N$ random matrix *M* is a sub-Gaussian random matrix, if

15 $${\rm{\Pr }}\,(|{M}_{j,k}|\ge \kappa )\le {C}_{1}{e}^{-{C}_{2}{\kappa }^{2}}$$

for $\forall \kappa  > 0$, where ${M}_{j,k}$ is the (*j*, *k*)-th element of *M*, $j\in [M]$, $k\in [N]$, where

16 $${C}_{1},{C}_{2} > 0$$

are sub-Gaussian parameters.

## Methods

### System Model

#### **Theorem 1**

(*Dense measurement*). *A QG structure with unitary* $U(\overrightarrow{\theta })={U}_{B}U(\overrightarrow{\theta ^{\prime} })$, *where the unitary sets an arbitrary computational basis B for an n*-*length input* $|X\rangle$ *as* ${U}_{B}|X\rangle =|S\rangle$, *such that* ${L}_{0}(S)\le K$, $K\ll n$, *holds for the L*_0_-*norm of S*, *where S is a classical representation of* $|S\rangle$, *while* $U(\overrightarrow{\theta ^{\prime} })$ *is the actual setting of the unitaries of QG at* $|S\rangle$ *and with a M*_*r*_ *random measurement operator*, allows the determination of the global optimal output z* *and global optimal estimate* $C({z}^{\ast })$ *at a particular objective function C as* $\varepsilon ={\rm{\Pr }}({\delta }_{K}\ge \chi )$ *holds*, *where δ*_*K*_ *and* $\chi$ *are constants depending on* ${\mathscr{Q}}= {\mathcal M} U(\overrightarrow{\theta ^{\prime} })$, *where* ${\mathcal M} =({M}_{r}^{(1)},\ldots ,{M}_{r}^{(R)})$ *and* ${M}_{r}^{(m)}$ *is the measurement operator of the m*-*th dense measurement round* $m=1,\ldots ,R$.

**Proof**. First, we rewrite (2) as

17 $$U(\overrightarrow{\theta })={U}_{B}U(\overrightarrow{\theta ^{\prime} }),$$

where *U*_*B*_ is a unitary that sets a computational basis *B* and $U(\overrightarrow{\theta ^{\prime} })$ is a unitary operation that sets the unitaries, such that

18 $$\begin{array}{rcl}{U}_{B}U(\overrightarrow{\theta ^{\prime} })\,{({U}_{B}U(\overrightarrow{\theta ^{\prime} }))}^{\dagger } & = & {U}_{B}U(\overrightarrow{\theta ^{\prime} }){U}_{B}^{\dagger }{(U(\overrightarrow{\theta ^{\prime} }))}^{\dagger }\\  & = & {U}_{B}U(\overrightarrow{\theta ^{\prime} })\,{(U(\overrightarrow{\theta ^{\prime} }))}^{\dagger }{U}_{B}^{\dagger }\\  & = & {U}_{B}I{U}_{B}^{\dagger }\\  & = & {U}_{B}{U}_{B}^{\dagger }=I,\end{array}$$

where *I* is the identity and $\overrightarrow{\theta ^{\prime} }$ is the *L*-dimensional vector of the gate parameters of $U(\overrightarrow{\theta ^{\prime} })$. Applying the unitary *U*_*B*_ on input system $|X\rangle$ yields the *n*-length quantum system $|S\rangle =|{s}_{1},\ldots ,{s}_{n}\rangle$,

19 $$|S\rangle ={U}_{B}|X\rangle ,$$

where the computational basis *B* for *U*_*B*_ in (17) is selected such that for the *L*_0_-norm of *S* the following relation holds

20 $${L}_{0}(S)={\Vert S\Vert }_{0}\le K,$$

where

21 $$S=BX$$

is a classical representation of $|S\rangle$, *X* is a classical representation of $|X\rangle$ and $K\ll n$. Therefore, *B* can be an arbitrary computational basis for which (20) holds at a given (21) (For example, if *B* is the Fourier basis, then *U*_*B*_ realizes a quantum Fourier transform).

The output of *QG* at (17) and (19) is therefore written as

22 $$\begin{array}{rcl}U(\overrightarrow{\theta })|X\rangle  & = & {U}_{B}U\,(\overrightarrow{\theta ^{\prime} })\,|X\rangle \\  & = & U\,(\overrightarrow{\theta ^{\prime} })\,({U}_{B}|X\rangle )\\  & = & U\,(\overrightarrow{\theta ^{\prime} })\,|S\rangle \\  & = & |G\rangle \\  & = & |{g}_{1},\ldots ,{g}_{n}\rangle ,\end{array}$$

whose state is measured by an *M*_*r*_ random measurement operator, defined as an *n*-bit length vector

23 $${M}_{r}={({b}_{1}{M}_{B},\ldots ,{b}_{n}{M}_{B})}^{T},$$

where *b*_*i*_ is a random variable,

24 $${b}_{i}=\{\begin{array}{ll}0, & {\rm{with}}\,\Pr (0)=0.5\\ 1, & {\rm{with}}\,{\rm{\Pr }}(1)=0.5\end{array},$$

associated with the measurement of the *i*-th quantum system $|{g}_{i}\rangle$ of $|G\rangle$ in (22), and *M*_*B*_ is a measurement in the computational basis *B*.

Thus, the measurement of the *i*-th quantum system $|{g}_{i}\rangle$ of $|G\rangle$ is defined via the following rule:

25 $${b}_{i}{M}_{B}=\{\begin{array}{ll}0, & {\rm{if}}\,{b}_{i}=0,\\ {M}_{B}, & {\rm{if}}\,{b}_{i}=1\end{array}.$$

In other words, the measurement result ${M}_{r}(|{g}_{i}\rangle )$ is kept only if ${b}_{i}=1$ in (23); otherwise, the measurement result is discarded and replaced by a zero element. This results output *y*_*i*_, as

26 $${y}_{i}=\{\begin{array}{ll}0, & {\rm{if}}\,{b}_{i}=0,\\ {M}_{B}(|{g}_{i}\rangle ) & {\rm{if}}\,{b}_{i}=1\end{array}.$$

This measurement strategy defines *M*_*r*_ (23) as a random Bernoulli vector^47–50^. Then, the *n*-bit length output *Y*, is as

27 $$\begin{array}{rcl}Y & = & {M}_{r}\,(|G\rangle )\\  & = & {M}_{r}U\,(\overrightarrow{\theta ^{\prime} })\,|S\rangle \\  & = & {M^{\prime} }_{r}\,|S\rangle \\  & = & {\beta }_{C}{\rm{\Lambda }}\\  & = & {\beta ^{\prime} }_{C}S,\end{array}$$

where $${M^{\prime} }_{r}$$ is

28 $${M^{\prime} }_{r}={M}_{r}U(\overrightarrow{\theta ^{\prime} })$$

while *β*_*C*_ is an *n*-length classical vector formulated via the *b*_*i*_ bits of (24) as

29 $${\beta }_{C}={({b}_{1},\ldots ,{b}_{n})}^{T},$$

and

30 $${\beta ^{\prime} }_{C}={\beta }_{C}U(\overrightarrow{\theta ^{\prime} }),$$

and $${\rm{\Lambda }}$$ is

31 $${\rm{\Lambda }}=U(\overrightarrow{\theta ^{\prime} })S.$$

As follows, applying *M*_*r*_ (23) on $|G\rangle$ (22) is equivalent to applying ${M^{\prime} }_{r}$ (28) on the computational basis state $|S\rangle$ (19).

As (27) is determined via (23), the goal is to determine *C*(*z*) at a particular objective function *C* via a post-processing ${\mathscr{P}}$.

First, from *Y* (27), the computational basis vector *S* can be recovered as $\tilde{S}$ via ${\mathscr{P}}$, as a minimization^50^,

32 $$\tilde{S}=\mathop{{\rm{\arg }}\,{\rm{\min }}}\limits_{S}\,{L}_{1}(S)$$

such that

33 $$Y={\beta ^{\prime} }_{C}S$$

where *L*_1_ is the *L*_1_-norm. The ${\mathscr{P}}$ unit utilizes a basis pursuit algorithm^47–50^ for the *L*_1_-minimization in (32). Then, using (32), $\tilde{{\rm{\Lambda }}}$ is defined as

34 $$\tilde{{\rm{\Lambda }}}=U(\overrightarrow{\theta ^{\prime} })\tilde{S}.$$

Thus, from (34), the output vector *z* is evaluated as

35 $$\begin{array}{rcl}z & = & {B}^{-1}{\mathscr{P}}(Y)\\  & = & {B}^{-1}(\tilde{{\rm{\Lambda }}})\\  & = & U(\overrightarrow{\theta })\tilde{X},\end{array}$$

where ${\mathscr{P}}(Y)$ is the post-processing (32) applied on *Y*, *B*^−1^ is the inverse basis transformation and $\tilde{X}$ is a classical representation of $|X\rangle$. As follows, from (35), the *C*(*z*) estimate yields

36 $$C(z)=C({B}^{-1}{\mathscr{P}}(Y)).$$

Then, assume that the procedure repeats for *R* rounds. The *R* rounds of dense measurement are defined via an $n\times R$ measurement matrix ${\mathcal M}$ as

37 $$ {\mathcal M} =({M}_{r}^{(1)},\ldots ,{M}_{r}^{(R)}),$$

where ${M}_{r}^{(m)}$ is an *n*-size random measurement vector (23) of the *m*-th measurement round $m=1,\ldots ,R$, as

38 $${M}_{r}^{(m)}={({b}_{1}^{(m)}{M}_{B},\ldots ,{b}_{n}^{(m)}{M}_{B})}^{T},$$

where ${b}_{i}^{(m)}$ is the *i*-th bit of ${M}_{r}^{(m)}$ defined via (24), and ${{M^{\prime} }_{r}}^{(m)}$ of the *m*-th round is

39 $${{M^{\prime} }_{r}}^{(m)}={M}_{r}^{(m)}U(\overrightarrow{\theta ^{\prime} }),$$

and ${{\beta ^{\prime} }_{C}}^{(m)}$ of the *m*-th round is

40 $${{\beta ^{\prime} }_{C}}^{(m)}={\beta }_{C}^{(m)}U(\overrightarrow{\theta ^{\prime} }),$$

where

41 $${\beta }_{C}^{(m)}={({b}_{1}^{(m)},\ldots ,{b}_{n}^{(m)})}^{T}.$$

For the *R* rounds, define the $n\times R$ orthogonal matrix ${\mathscr{Q}}$ as

42 $${\mathscr{Q}}= {\mathcal M} U(\overrightarrow{\theta ^{\prime} })=({{M^{\prime} }_{r}}^{(1)},\ldots ,{{M^{\prime} }_{r}}^{(R)}),$$

and the measurement output matrix *Y*^*R*^ as

43 $${Y}^{R}={\mathscr{Q}}|S\rangle =({Y}^{(1)},\ldots ,{Y}^{(R)}),$$

where *Y*^(*m*)^ is the measurement result vector (33) of the *m*-th round.

The problem is therefore to find the optimal value of *R*, such that the total error probability at the end of *R* rounds

44 $${\rm{\Pr }}(z\ne {z}^{\ast })=\xi $$

picks up a given arbitrary value $\xi$ that is determined via the success of the *L*_1_ minimization (32) in the ${\mathscr{P}}$ unit.

After some argumentations on the probability distribution of ${\mathscr{Q}}$ (42), at *R* measurement rounds a concentration relation can be written as

45 $$\begin{array}{l}{\rm{\Pr }}(|{({L}_{2}({\mathscr{Q}}|S\rangle ))}^{2}-{\ell }^{2}(|S\rangle )|\ge \kappa ({\ell }^{2}(|S\rangle )))\\ \begin{array}{rcl} & = & {\rm{\Pr }}(|{({L}_{2}( {\mathcal M} |G\rangle ))}^{2}-{\ell }^{2}(|G\rangle )|\ge \kappa ({\ell }^{2}(|G\rangle )))\\  & \le  & 2\,\exp (\,-\,c{\kappa }^{2}R),\end{array}\end{array}$$

where *c* is a constant depending on the sub-Gaussian parameters ${C}_{1}$, ${C}_{2} > 0$ (see Section 4.1) of the sub-Gaussian matrix ${\mathscr{Q}}$ (42), *L*_2_ is the Euclidean norm and ${\ell }^{2}$ is the ${\ell }^{2}$-norm of a quantum system, ${\ell }^{2}(|\psi \rangle )=\sqrt{{\sum }_{x}\,{|\psi (x)|}^{2}}=1$, where ${|\psi (x)|}^{2}={\rm{\Pr }}(x)$ and $\sqrt{\int \,{\rm{\Pr }}(x)dx}=\sqrt{\int \,{|\psi (x)|}^{2}dx}=1$, while $\kappa$ is $\kappa \in (0,1)$.

By theory, the *K*-th restricted isometry constant^47–50^ ${\delta }_{K}={\delta }_{K}({\mathscr{Q}})$ of matrix ${\mathscr{Q}}$ is the smallest $\chi \ge 0$ such that

46 $$(1-\chi ){\ell }^{2}(|S\rangle )\le {({L}_{2}({\mathscr{Q}}|S\rangle ))}^{2}\le (1+\chi ){\ell }^{2}(|S\rangle ),$$

for $$\forall S$$ where $${L}_{0}(S)\le K$$.

Then, for a given $\chi$, the restricted isometry constant^47–50^ *δ*_*K*_ of ${\mathscr{Q}}= {\mathcal M} U(\overrightarrow{\theta ^{\prime} })$ satisfies relation ${\delta }_{K} < \chi$ with probability

47 $${\rm{\Pr }}({\delta }_{K} < \chi )=1-\varepsilon $$

where $\varepsilon \in (0,1)$, if *R* is selected as

48 $$R=A\frac{1}{{\chi }^{2}}(K(9+2\,\mathrm{log}(\frac{n}{K}))+2\,\mathrm{log}(2(\frac{1}{\varepsilon }))),$$

where

49 $$A=\frac{2}{3c}.$$

The motivation for the selection of *R* is as follows. The value of *R* in (48) guarantees that the relation ${\delta }_{K} < \chi$ holds with probability $1-\varepsilon$, as it is given in (47). If *R* is greater than (48), then ${\rm{\Pr }}({\delta }_{K} < \chi ) > 1-\varepsilon$, while if *R* is lower than the value given in (48), then ${\rm{\Pr }}({\delta }_{K} < \chi ) < 1-\varepsilon$. As a corollary, the lowest value of *R* to satisfy the relation ${\delta }_{K} < \chi$ with probability at least $1-\varepsilon$, is as given in (48). To prove (48), express *δ*_*K*_ via (46) as

50 $${\delta }_{K}=\mathop{{\rm{\sup }}}\limits_{\Upsilon \subset [n],|\Upsilon |=K}\,{L}_{2}({{\mathscr{Q}}}_{\Upsilon }^{\ast }{{\mathscr{Q}}}_{\Upsilon }-I),$$

where $\Upsilon$ is subset, ${{\mathscr{Q}}}_{\Upsilon }$ is a submatrix, *I* is the identity matrix, $|\Upsilon |$ is the cardinality of subset $\Upsilon$ and $[n]=\{1,\ldots ,n\}$ is the set of natural numbers not exceeding *n*.

The formula of (50) is equivalent to (46), since (46) can be rewritten as

51 $$|{({L}_{2}({{\mathscr{Q}}}_{\Upsilon }|S^{\prime} \rangle ))}^{2}-{\ell }^{2}(|S^{\prime} \rangle )|\le \chi {\ell }^{2}(|S^{\prime} \rangle )$$

for $\forall \Upsilon \subset [n]$, $|\Upsilon |\le K$ and $S^{\prime} \subset S$. Let

52 $${Y}_{\Upsilon }={{\mathscr{Q}}}_{\Upsilon }|S^{\prime} \rangle ,$$

and

53 $${Z}_{\Upsilon }=H|S^{\prime} \rangle ,$$

where *H* is a Hermitian matrix,

54 $$H={{\mathscr{Q}}}_{\Upsilon }^{\ast }{{\mathscr{Q}}}_{\Upsilon }-I,$$

then

55 $$\begin{array}{rcl}{({L}_{2}({Y}_{\Upsilon }))}^{2}-{\ell }^{2}(|S^{\prime} \rangle ) & = & \langle {Y}_{\Upsilon },{Y}_{\Upsilon }\rangle -\langle S^{\prime} ,S^{\prime} \rangle \\  & = & \langle {Z}_{\Upsilon },S^{\prime} \rangle \end{array}$$

Therefore, ${L}_{2}(H)$ can be expressed as a maximization

56 $${L}_{2}(H)=\mathop{{\rm{\max }}}\limits_{S^{\prime} }\frac{\langle {Z}_{\Upsilon },S^{\prime} \rangle }{{\ell }^{2}(|S^{\prime} \rangle )}$$

that leads to relation

57 $$\mathop{{\rm{\max }}}\limits_{\Upsilon \subset [n],|\Upsilon |=K}\,{L}_{2}(H)\le \chi .$$

Then, the union bound takes over all $$(\begin{array}{c}n\\ K\end{array})$$ subsets $\Upsilon \subset [n]$ of cardinality *K*, yields the relation of

58 $$\begin{array}{rcl}{\rm{\Pr }}({\delta }_{K}\ge \chi ) & \le  & \sum _{{{\rm{\sup }}}_{\Upsilon \subset [n],|\Upsilon |=K}}\,{\rm{\Pr }}({L}_{2}(H)\ge \chi )\\  & \le  & 2(\begin{array}{c}n\\ K\end{array}){(1+\tfrac{2}{{\rm{\Omega }}})}^{K}\,\exp (\,-\,c{\chi }^{2}{(1-2{\rm{\Omega }})}^{2}R)\\  & \le  & 2{(\tfrac{en}{K})}^{K}{(1+\tfrac{2}{{\rm{\Omega }}})}^{K}\,\exp (\,-\,c{\chi }^{2}{(1-2{\rm{\Omega }})}^{2}R),\end{array}$$

where we used that for integers $m\ge k > 0$, $${(\frac{m}{k})}^{k}\le (\begin{array}{c}m\\ k\end{array})\le {(\frac{em}{k})}^{k}$$ by theory^47–50^.

It can be verified that in (58) for $\Upsilon \subset [n]$ with $|\Upsilon |=K$, the relation

59 $${\rm{\Pr }}({L}_{2}(H) < \chi )=1-\varepsilon $$

holds, if

60 $$R=\frac{2}{3c{\chi }^{2}}(7K+2\,\mathrm{log}(2(\frac{1}{\varepsilon }))),$$

since for

61 $$\kappa =(1-2{\rm{\Omega }})\chi $$

it can be verified that

62 $${\rm{\Pr }}({L}_{2}(H)\ge \chi )\le 2{(1+\frac{2}{{\rm{\Omega }}})}^{K}\,\exp (\,-\,c{(1-2{\rm{\Omega }})}^{2}{\chi }^{2}R).$$

Thus, (59) is satisfied only if

63 $$R=\frac{1}{c{(1-2{\rm{\Omega }})}^{2}{\chi }^{2}}(\mathrm{log}(1+\frac{2}{{\rm{\Omega }}})K+\,\mathrm{log}(2(\frac{1}{\varepsilon }))).$$

Then, setting ${\rm{\Omega }}$ in (63) to

64 $${\rm{\Omega }}=\frac{2}{{e}^{3.5}-1}$$

so that

65 $$\frac{1}{{(1-2{\rm{\Omega }})}^{2}}\le \frac{4}{3}$$

and

66 $$\mathrm{log}(1+\frac{2}{{\rm{\Omega }}})\frac{1}{{(1-2{\rm{\Omega }})}^{2}}\le \frac{14}{3},$$

yields (60)^50^.

Note that it also can be shown that for ${\rm{\Omega }}\in (0,0.5)$ in (64), there exists a finite subset ${\rm{\Gamma }}$ of a unit ball ${ {\mathcal B} }_{\Upsilon }=\{X,{\rm{supp}}X\subset \Upsilon ,{\ell }^{2}(|X\rangle )\le 1\}$ such that $|{\rm{\Gamma }}|$ is

67 $$|{\rm{\Gamma }}|\le {(1+\frac{2}{{\rm{\Omega }}})}^{K}$$

and

68 $$\mathop{{\rm{\min }}}\limits_{x\in {\rm{\Gamma }}}\,{L}_{2}(z-x)\le {\rm{\Omega }}$$

for $\forall z\in { {\mathcal B} }_{\Upsilon }$, such that for $x\subset {\rm{\Gamma }}$

69 $$\begin{array}{l}{\rm{\Pr }}(|{({L}_{2}({\mathscr{Q}}|x\rangle ))}^{2}-{\ell }^{2}(|x\rangle )|\ge \kappa ({\ell }^{2}(|x\rangle )))\\ \begin{array}{rcl} & \le  & \sum _{x\in {\rm{\Gamma }}}\,\Pr (|{({L}_{2}({\mathscr{Q}}|x\rangle ))}^{2}-{\ell }^{2}(|x\rangle )|\ge \kappa ({\ell }^{2}(|x\rangle )))\\  & \le  & 2|{\rm{\Gamma }}|\,\exp (\,-\,c{\kappa }^{2}R)\\  & \le  & 2{(1+\tfrac{2}{{\rm{\Omega }}})}^{K}\,\exp (\,-\,c{\kappa }^{2}R),\end{array}\end{array}$$

and

70 $$\begin{array}{l}{\rm{\Pr }}(|{({L}_{2}({\mathscr{Q}}|x\rangle ))}^{2}-{\ell }^{2}(|x\rangle )| < \kappa ({\ell }^{2}(|x\rangle )),\forall x\subset {\rm{\Gamma }})\\ \begin{array}{rcl} & = & 1-2{(1+\tfrac{2}{{\rm{\Omega }}})}^{S}\,\exp (\,-\,c{\kappa }^{2}R).\end{array}\end{array}$$

By finding the values of ${\rm{\Omega }}$ and $\kappa$, the relation

71 $$|{({L}_{2}({\mathscr{Q}}|z\rangle ))}^{2}-{\ell }^{2}(|z\rangle )|={L}_{2}(H)\le \chi $$

can be satisfied for $\forall z\in { {\mathcal B} }_{\Upsilon }$.

It can be proven at *H* (54) and

72 $$W=H|x\rangle ,$$

for $\forall x\subset {\rm{\Gamma }}$ that the relation

73 $$|\langle W,x\rangle | < \kappa ,$$

holds. Thus, for a given *z* and $x\subset {\rm{\Gamma }}$, such that ${L}_{2}(z-x)\le {\rm{\Omega }}\le \frac{1}{2}$,

74 $$\begin{array}{rcl}|\langle V,z\rangle | & = & |\langle W,x\rangle +\langle D,z-x\rangle |\\  & \le  & |\langle W,x\rangle |+|\langle D,z-x\rangle |\\  &  <  & \kappa +{L}_{2}(H)\sqrt{{\ell }^{2}(|z+x\rangle )}\sqrt{{\ell }^{2}(|z-x\rangle )}\\  & \le  & \kappa +2{L}_{2}(H){\rm{\Omega }}\end{array}$$

where

75 $$V=H|z\rangle ,$$

and

76 $$D=H|z+x\rangle .$$

Then, a maximization over $\forall z\in { {\mathcal B} }_{\Upsilon }$ yields

77 $${L}_{2}(H) < \kappa +2{L}_{2}(H){\rm{\Omega }}.$$

Thus,

78 $${L}_{2}(H)\le \frac{\kappa }{1-2{\rm{\Omega }}}.$$

As follows, there exists (61) such that ${L}_{2}(H) < \chi$ holds, and combining it with (70) verifies the relation of (62).

To conclude the results, setting ${\rm{\Omega }}$ in (58) with equality in (64) leads to ${\delta }_{K} < \chi$ with probability ${\rm{\Pr }}({\delta }_{K} < \chi )=1-\varepsilon$, as the *R* value of measurement rounds is

79 $$\begin{array}{rcl}R & = & \tfrac{1}{c{\chi }^{2}}(\tfrac{4}{3}K\,\mathrm{log}(\tfrac{10n}{K})+\tfrac{14}{3}K+\tfrac{4}{3}\,\mathrm{log}(2(\tfrac{1}{\varepsilon })))\\  & = & \tfrac{2}{3c}\tfrac{1}{{\chi }^{2}}(K(9+2\,\mathrm{log}(\tfrac{n}{K}))+2\,\mathrm{log}(2(\tfrac{1}{\varepsilon }))).\end{array}$$

◼

Note that if *R* is selected to be greater than (79), the probability is increased to ${\rm{\Pr }}({\delta }_{K} < \chi ) > 1-\varepsilon$.

### Dense Measurement Rounds in Gate-Model Quantum Computers

#### Arbitrary Unitary Sequences

The next theorem reveals that the number *R* of dense measurement rounds can be used to determine z* with an error probability $\zeta ={n}^{-{\mathrm{log}}^{3}(n)}$, such that *R* depends only on the properties of the unitaries, while it does not depend directly on the actual $\zeta$.

##### **Theorem 2**

 (*Dense measurements at a* $U(\overrightarrow{\theta })$ *quantum gate structure*). *For an arbitrary unitary U*_*B*_ *in* $U(\overrightarrow{\theta })={U}_{B}U(\overrightarrow{\theta ^{\prime} })$ *with* ${L}_{0}(S)\le K$, the global optimal z* *and estimate* $C({z}^{\ast })$ *can be determined via* $R=\alpha {Z}^{2}K\,{\mathrm{log}}^{4}(n)$ *dense measurement rounds*, *with probability* ${\rm{\Pr }}({z}^{\ast })={\rm{\Pr }}(C({z}^{\ast }))=1-{n}^{-{\mathrm{log}}^{3}(n)}$, *where* $Z\ge \sqrt{n}\mathop{{\rm{\max }}}\limits_{k,q\in [n]}|U({\overrightarrow{\theta }}_{q,k})|$, $U({\overrightarrow{\theta }}_{q,k})$ *is the q*-*th element of the k*-*th column of* $U(\overrightarrow{\theta })$, *while* $\alpha  > 0$ *is a constant*.

**Proof**. Let assume that $U(\overrightarrow{\theta })$ can be decomposed as ${U}_{B}U(\overrightarrow{\theta ^{\prime} })$, and the following bound can be formulated for the entries of $U(\overrightarrow{\theta })$,

80 $$\mathop{{\rm{\max }}}\limits_{k,q\in [n]}|U({\overrightarrow{\theta }}_{q,k})|\le \frac{Z}{\sqrt{n}},$$

where $U({\overrightarrow{\theta }}_{q,k})$ is the *q*-th element of the *k*-th column of $U(\overrightarrow{\theta })$, and

81 $$|U({\overrightarrow{\theta }}_{q,k})|=\sqrt{U({\overrightarrow{\theta }}_{q,k})\,{(U({\overrightarrow{\theta }}_{q,k}))}^{\ast }}.$$

Let assume that the size of $U(\overrightarrow{\theta })$ is $n\times n$, with columns *u*_*k*_, $k=1,\ldots ,n$. Then let *v*_*k*_ be the normalization of *u*_*k*_ as

82 $${v}_{k}=\sqrt{n}{u}_{k},$$

where the normalized columns form an orthonormal system, and let ${\phi }_{kl}$ be the inner product of two normalized columns *v*_*k*_ and *v*_*l*_, as

83 $${\phi }_{kl}=\langle \frac{1}{\sqrt{n}}{v}_{k},\frac{1}{\sqrt{n}}{v}_{l}\rangle =\langle {u}_{k},{u}_{l}\rangle ,$$

that can be rewritten as

84 $${\phi }_{kl}=\frac{1}{n}\,\sum _{q=1}^{n}\,\sqrt{n}U({\overrightarrow{\theta }}_{q,k})\sqrt{n}{U}^{\dagger }({\overrightarrow{\theta }}_{q,l})=\frac{1}{n}\,\sum _{q=1}^{n}\,\sqrt{n}{u}_{k,q}\sqrt{n}{u}_{l,q}^{\dagger },$$

where ${u}_{i,j}=U({\overrightarrow{\theta }}_{j,i})$ and ${v}_{i,j}=\sqrt{n}U({\overrightarrow{\theta }}_{j,i})$. Therefore, in (84), the sum operator runs over the *n* elements of the *k*-th column of unitary $U(\overrightarrow{\theta })$, and the *n* elements of the *l*-th column of ${U}^{\dagger }(\overrightarrow{\theta })$, respectively.

Then, at *U*_*B*_ and $U(\overrightarrow{\theta ^{\prime} })$, some argumentations on bounded orthonormal systems straightforwardly yields the boundedness condition^50^

85 $$Z\ge \mathop{{\rm{\max }}}\limits_{k,q\in [n]}|\langle {b}_{q},{u^{\prime} }_{k}\rangle |,$$

where *b*_*q*_ is the *q*-th column of *U*_*B*_.

Then, for the maximal entry of $U({\overrightarrow{\theta }}_{q,k})$, a bound can be established via the normalized columns, as

86 $$\begin{array}{rcl}Z\ge \mathop{{\rm{\max }}}\limits_{k,q\in [n]}\,|{v}_{k,q}| & = & \mathop{{\rm{\max }}}\limits_{k,q\in [n]}\,|\sqrt{n}{u}_{k,q}|\\  & = & \sqrt{n}\mathop{{\rm{\max }}}\limits_{k,q\in [n]}\,|{u}_{k,q}|=\sqrt{n}\mathop{{\rm{\max }}}\limits_{k,q\in [n]}\,|U({\overrightarrow{\theta }}_{q,k})|.\end{array}$$

As follows, the bounds in (85) and (86) are equivalent to (80).

Then, by introducing a projector ${{\mathscr{P}}}_{{Q}_{R}}$ that selects a subset of $U(\overrightarrow{\theta })$ in the *R* rounds, the ${\mathcal M}$ (37) measurement operator applied on a unitary $U(\overrightarrow{\theta })$ can be rewritten as

87 $$ {\mathcal M} ={{\mathscr{P}}}_{{Q}_{R}}(U(\overrightarrow{\theta })),$$

where ${Q}_{R}\subset [n]$ is a subset of *R* elements selected uniform at random from all subsets of [*n*] of cardinality *R*, $|{Q}_{R}|=R$,

88 $${Q}_{R}=\{{q}_{1},\ldots ,{q}_{R}\}.$$

As follows, the *Y*^*R*^ (43) measurement result can be rewritten as

89 $${Y}^{R}={{\mathscr{P}}}_{{Q}_{R}}(U(\overrightarrow{\theta }))|X\rangle ={{\mathscr{P}}}_{{Q}_{R}}(U(\overrightarrow{\theta ^{\prime} }){U}_{B})|X\rangle ={{\mathscr{P}}}_{{Q}_{R}}(U(\overrightarrow{\theta ^{\prime} }))|S\rangle .$$

It is required to verify that the $\xi$ error probability (44) at a projector ${{\mathscr{P}}}_{Q}$ in (87) is bounded by an ${\xi }^{\ast }$ error probability associated with the selection of rows uniformly and independently at random from $U(\overrightarrow{\theta })$^50^.

Thus, we define set ${Q^{\prime} }_{R}\subset [n]$ with the same cardinality as (88), such that its elements are selected independently and uniformly at random from [*n*], $|{Q^{\prime} }_{R}|=R$

90 $${Q^{\prime} }_{R}=\{{q^{\prime} }_{1},\ldots ,{q^{\prime} }_{R}\}.$$

Then, let ${Q}_{k}\subset [n]$ be a subset of $k\le R$ selected uniform at random from all subsets of [*n*] of cardinality *k*, $|{Q}_{k}|=k$,

91 $${Q}_{k}=\{{q}_{1},\ldots ,{q}_{k}\}.$$

For any subset $Q\in [n]$, we define a failure event ${\mathcal E} (Q)$ as

92 $$ {\mathcal E} (Q)\equiv \{\tilde{S}\ne \mathop{{\rm{\arg }}\,{\rm{\min }}}\limits_{S}\,{L}_{1}(S),\,{\rm{s}}.{\rm{t}}.\,Y={{\mathscr{P}}}_{Q}(U(\overrightarrow{\theta ^{\prime} }))|S\rangle ,\,{\rm{for}}\,\forall |S\rangle \},$$

i.e., the event that the *L*_1_-minimization (i.e,. a basis pursuit algorithm in ${\mathscr{P}}$) allows no to determine every *S* from (89) on *Q* (Note that the success probability of an *L*_1_-minimization in ${\mathscr{P}}$ to determine *S* is independent from the normalization of the measurement operator).

It can be verified, that for $Q\subset \tilde{Q}$,

93 $$ {\mathcal E} (\tilde{Q})\subset  {\mathcal E} (Q),$$

and for $$k\le R$$,

94 $${\rm{\Pr }}( {\mathcal E} ({Q}_{R}))=\xi \le {\rm{\Pr }}( {\mathcal E} ({Q}_{k}))={\xi }_{k},$$

and if $|{Q^{\prime} }_{R}|=k$ holds for $k\le R$, then

95 $${\mathscr{D}}({Q^{\prime} }_{R})={\mathscr{D}}({Q}_{k}),$$

where ${\mathscr{D}}$(·) is the distribution.

Therefore, the

96 $${\rm{\Pr }}( {\mathcal E} ({Q^{\prime} }_{R}))={\xi }^{\ast }$$

probability of event ${\mathcal E} ({Q^{\prime} }_{R})$ at $|{Q^{\prime} }_{R}|=k$ is as

97 $$\begin{array}{rcl}{\xi }^{\ast } & = & \sum _{k=1}^{R}\,{\rm{\Pr }}( {\mathcal E} ({Q^{\prime} }_{R})|(|{Q^{\prime} }_{R}|=k))\,{\rm{\Pr }}(|{Q^{\prime} }_{R}|=k)\\  & = & \sum _{k=1}^{R}\,{\xi }_{k}\,{\rm{\Pr }}(|{Q^{\prime} }_{R}|=k)\ge \xi \,\sum _{k=1}^{R}\,{\rm{\Pr }}(|{Q^{\prime} }_{R}|=k)\\  & = & \xi ,\end{array}$$

thus the error probability $\xi$ is bounded by ${\xi }^{\ast }$.

As follows, using projector ${{\mathscr{P}}}_{Q}$ in (87), an *L*_1_-minimization (basis pursuit)^47–50^ in the ${\mathscr{P}}$ post-processing phase allows to determine the global optimal z* from (89), with probability

98 $${\rm{\Pr }}({z}^{\ast })=1-{n}^{-{\mathrm{log}}^{3}(n)},$$

as

99 $$R=\alpha {Z}^{2}K\,{\mathrm{log}}^{4}(n)$$

holds, where $\alpha  > 0$, and $\xi$ is evaluated via (44) as

100 $$\xi ={\rm{\Pr }}(z\ne {z}^{\ast })={n}^{-{\mathrm{log}}^{3}(n)}.$$

The value determined for *R* in (99) is based on the following fact. It can be shown^50^, that at a particular $\alpha  > 0$ and $Z\ge 1$, there exists a constant *a*, $a\in (0,1)$, such that for a given *a*, the relation

101 $${\delta }_{K}\le a$$

holds with probability (98), if

102 $$R\ge \tfrac{1}{{a}^{2}}\alpha {Z}^{2}K\,{\mathrm{log}}^{4}(n),$$

where

103 $${\delta }_{K}={\delta }_{K}(\tfrac{1}{\sqrt{R}}{\mathscr{Q}})$$

is the *K*-th restricted isometry constant of $\tfrac{1}{\sqrt{R}}{\mathscr{Q}}$, that is the smallest $a\ge 0$ such that

104 $$(1-a){\ell }^{2}(|S\rangle )\le {({L}_{2}(\tfrac{1}{\sqrt{R}}{\mathscr{Q}})|S\rangle ))}^{2}\le (1+a){\ell }^{2}(|S\rangle )$$

while ${\mathscr{Q}}$ is as given by (42); holds for $\forall S$, with ${L}_{0}(S)\le K$. Since for (99), $\alpha  > 0$ holds and $Z\ge 1$ is satisfied via (86), the result in (99) is straightforwardly follows from in (102) at

105 $$a=1.$$

Thus, at (99) dense measurement rounds the global optimal output z*

106 $${z}^{\ast }={B}^{-1}({{\rm{\Lambda }}}^{\ast }),$$

is yielded with probability

107 $${\rm{\Pr }}({z}^{\ast })=1-\xi ,$$

where ${{\rm{\Lambda }}}^{\ast }$ is the optimal $\tilde{{\rm{\Lambda }}}$ determined via ${\mathscr{P}}$. Therefore the *C*(z*) global optimal estimate of a particular objective function *C* can be determined with probability $Pr(C({z}^{\ast }))=Pr({z}^{\ast })$ as

108 $$C({z}^{\ast })=\mathop{{\rm{\max }}}\limits_{\forall m}\,C({z}_{m}),$$

where *C*(*z*_*m*_) is the estimate yielded in an *m*-th round, $m=1,\ldots ,R$, *z*_*m*_ is the recovered output vector in the *m*-th round, which concludes the proof. ◼

The steps of the dense measurement for an arbitrary *U*$(\overrightarrow{\theta })$ are summarized in Procedure 1.

**Procedure 1:**
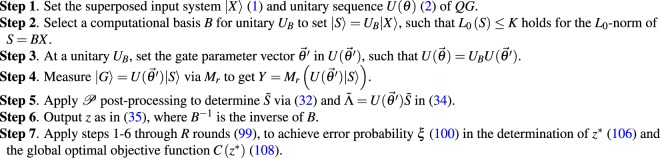
Dense Measurements at arbitrary unitary *U*$(\overrightarrow{\theta })$.

**Figure 1:**
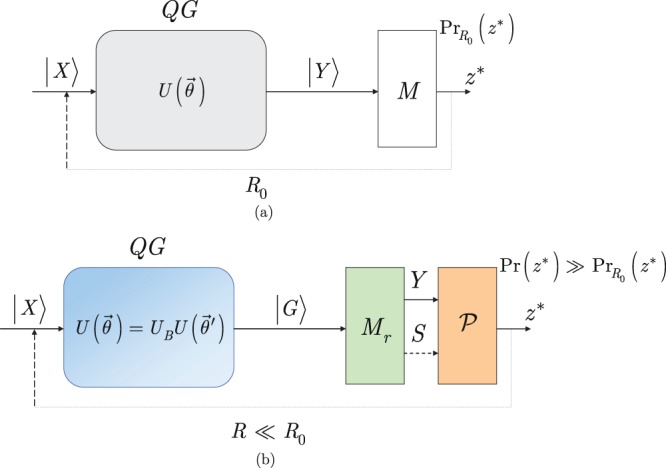
(**a**) A standard quantum measurement setting. The $|X\rangle$ superposed input quantum system is fed into the *QG* quantum gate structure. The *QG* structure realizes the unitary $U(\overrightarrow{\theta })$ and outputs $|Y\rangle =U(\overrightarrow{\theta })\,|X\rangle$. The output is measured by a standard (projective) measurement operator *M* in a computational basis, which results in an optimal output z* after *R*_0_ measurement rounds (depicted by the dashed line) with a success probability of ${{\rm{\Pr }}}_{{R}_{0}}({z}^{\ast })$. (**b**) The dense quantum measurement procedure. The $|X\rangle$ superposed input quantum system is fed into the *QG* quantum gate structure. The *QG* structure is set to realize the unitary $U(\overrightarrow{\theta })={U}_{B}U(\overrightarrow{\theta ^{\prime} })$. The *M*_*r*_ randomized measurement yields $Y={M}_{r}(U(\overrightarrow{\theta ^{\prime} })\,|S\rangle )$, which is post-processed along with $S=BX$ via unit ${\mathscr{P}}$. Unit ${\mathscr{P}}$ performs an *L*_1_-norm minimization and outputs the global optimal z* after $R\ll {R}_{0}$ rounds (depicted by the dashed line), with a high success probability of ${\rm{\Pr }}({z}^{\ast })\gg {{\rm{\Pr }}}_{{R}_{0}}({z}^{\ast })$.

Figure 1 depicts an application of dense quantum measurement in quantum computations. Figure 1(a) shows an arbitrary *QG* quantum circuit with a *U*$(\overrightarrow{\theta })$ sequence (where $\overrightarrow{\theta }$ sets the unitaries of the quantum circuit) and standard measurement *M* with *R*_0_ measurement rounds and success probability ${{\rm{\Pr }}}_{{R}_{0}}({z}^{\ast })$. Figure 1(b) shows dense quantum measurement in a general case *U*$(\overrightarrow{\theta })$, such that the *QG* structure is prepared to realize $U(\overrightarrow{\theta })={U}_{B}U(\overrightarrow{\theta ^{\prime} })$, where unitary *U*_*B*_ sets a computational basis as $|S\rangle ={U}_{B}|X\rangle$ with relation *L*_0_(*S*) for the *L*_0_-norm of *S* (*S* is a classical value resulting from the measurement), while *U*$(\overrightarrow{\theta ^{\prime} })$ is the actual setting of the unitaries of *QG* to provide output $|G\rangle =U(\overrightarrow{\theta ^{\prime} })|S\rangle$, such that $U(\overrightarrow{\theta ^{\prime} })\,|S\rangle =U(\overrightarrow{\theta })\,|X\rangle$. The output is measured via a randomized measurement *M*_*r*_. The measurement result is then post-processed via unit ${\mathscr{P}}$ to achieve $R\ll {R}_{0}$ and ${\rm{\Pr }}({z}^{\ast })\gg {{\rm{\Pr }}}_{{R}_{0}}({z}^{\ast })$.

#### Dense Measurements of Computational Basis Quantum States

The next theorem reveals that the number of dense measurement rounds can be reduced if the unitaries of the quantum circuit are set as $U(\overrightarrow{\theta })={U}_{B}$, i.e., if the output of the quantum circuit is a computational basis state $|S\rangle ={U}_{B}|X\rangle$.

##### **Theorem 3**

 (*Number of dense measurement rounds at* $U(\overrightarrow{\theta })={U}_{B}$). *At* $U(\overrightarrow{\theta })={U}_{B}$, the optimal z* *and C*(z*) *can be determined via* $R(\xi )={c}_{1}K\,\mathrm{log}(10n/K)+{c}_{2}\,\mathrm{log}(2/\xi )$ *dense measurement rounds*, *where* $\xi \in (0,1)$ is the error probability of z*, *while* ${c}_{1} > 0$ *and* ${c}_{2} > 0$ *are constants*, *that yields* $R={\mathscr{Q}}(K\,\mathrm{log}(\frac{10n}{K}))$, *with* $\xi =2\,\exp \,(\,-\,R)$.

**Proof**. In this setting, the unitaries of the *QG* quantum gate structure are set such that

109 $$U(\overrightarrow{\theta })={U}_{B}$$

holds, therefore the output of *QG* at an *n*-length input $|X\rangle$ is

110 $${U}_{B}|X\rangle =|S\rangle ,$$

i.e., it outputs an *n*-length computational basis quantum state $|S\rangle$, with relation ${L}_{0}(S)\le K$, $K\ll n$.

The *R* measurements are performed according to the $n\times R$ measurement matrix ${\mathcal M}$ as defined in (37).

Since $U(\overrightarrow{\theta })$ is set as given in (109), the measurement results of the *R* rounds formulate *R* dimensional output ${Y}^{R}=({Y}^{(1)},\ldots ,{Y}^{(R)})$ as

111 $${Y}^{R}= {\mathcal M} |S\rangle ,$$

where the output of the *m*-th round is

112 $${Y}^{(m)}={M}_{r}^{(m)}|S\rangle ={\beta }_{C}^{(m)}S,$$

where ${M}_{r}^{(m)}$ is as given in (38), while ${\beta }_{C}^{(m)}$ is as in (41).

It further can be verified that ${\mathcal M}$ is a sub-Gaussian random matrix, thus there exists a constant $\lambda  > 0$ depending only on the *C*_1_, *C*_2_ sub-Gaussian parameters (see Section 4.1) of ${\mathcal M}$ such that for a given $\chi$, the restricted isometry constant^47–50^ ${\delta }_{K}={\delta }_{K}( {\mathcal M} )$ of ${\mathcal M}$, satisfies relation ${\delta }_{K} < \chi$ with probability

113 $${\rm{\Pr }}({\delta }_{K} < \chi )=1-\varepsilon ,$$

if

114 $$R=\lambda \frac{1}{{\chi }^{2}}(K\,\mathrm{log}(10n/K)+\,\mathrm{log}(2/\varepsilon )),$$

where ${\delta }_{K}={\delta }_{K}( {\mathcal M} )$ is the *K*-th restricted isometry constant of ${\mathcal M}$, which is the smallest $\chi \ge 0$ such that

115 $$(1-\chi ){\ell }^{2}(|S\rangle )\le {({L}_{2}( {\mathcal M} |S\rangle ))}^{2}\le (1+\chi ){\ell }^{2}(|S\rangle ),$$

for $\forall S$, with ${L}_{0}(S)\le K$. Note, that at $\varepsilon =2\,\exp \,(\,-\,\frac{R}{{\chi }^{2}2\lambda })$, (114) picks up the value of $R=2\lambda \frac{1}{{\chi }^{2}}(K\,\mathrm{log}\,(\frac{10n}{K}))$.

Then, some argumentation on the *L*_1_-minimization based recovery via basis pursuit in the ${\mathscr{P}}$ unit, yields a condition for the 2*K*-th restricted isometry constant, *δ*_2*K*_ of ${\mathcal M}$ as

116 $${\delta }_{2K} < \frac{1}{3}.$$

The condition in (116) allows to determine any $\tilde{S}$ in the ${\mathscr{P}}$ post-processing as a unique solution of

117 $$\tilde{S}=\mathop{{\rm{\arg }}\,{\rm{\min }}}\limits_{S}\,{L}_{1}(S)$$

subject to

118 $${Y}^{R}={\beta }_{C}^{R}S,$$

with success probability

119 $${\rm{\Pr }}(\tilde{S})=1-\varepsilon =1-\xi ,$$

where ${\beta }_{C}^{R}$ is as

120 $${\beta }_{C}^{R}=({\beta }_{C}^{(1)},\ldots ,{\beta }_{C}^{(R)}),$$

such that for every *S* there exists a unique solution of (117).

Thus, in an *m*-th measurement round, output vector *z*_*m*_ is evaluated as

121 $$\begin{array}{rcl}{z}_{m} & = & {\mathscr{P}}({Y}^{(m)})\\  & = & \tilde{S}\\  & = & {U}_{B}\tilde{X}\\  & = & U(\overrightarrow{\theta })\tilde{X},\end{array}$$

where *Y*^(*m*)^ is given in (112).

Since (116) puts a strict bound on $\chi$, it allows us to rewrite (114) at a particular

122 $$\xi =\varepsilon $$

as

123 $$R(\xi )={c}_{1}K\,\mathrm{log}(\frac{10n}{K})+{c}_{2}\,\mathrm{log}(\frac{2}{\xi }),$$

where *c*_1_, ${c}_{2} > 0$ are constants depend only on the *C*_1_, *C*_2_ sub-Gaussian parameters (see Section 4.1) of ${\mathcal M}$.

Therefore, the global optimal z* can be determined via *R* rounds via ${\mathscr{P}}$, without loss of generality as

124 $$R={\mathscr{O}}(\gamma K\,\mathrm{log}(\frac{10n}{K})),$$

where $\gamma  > 0$ is a constant, that yields *C*(z*) via (108). Thus, at $\gamma =1$, the success probability is

125 $$\begin{array}{rcl}{\rm{\Pr }}({z}^{\ast }) & = & {\rm{\Pr }}(C({z}^{\ast }))\\  & = & 1-2\,\exp (\,-\,R)=1-\xi ,\end{array}$$

that concludes the proof. ◼

The steps of the dense measurement for an computational basis quantum states are summarized in Procedure 2.

**Procedure 2:**
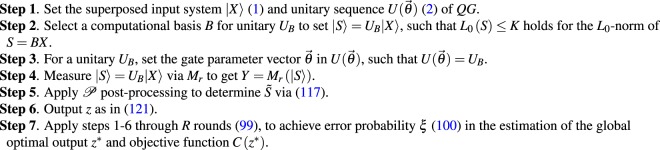
Dense measurements at $U(\overrightarrow{\theta })={U}_{B}$.

### Ethics statement

This work did not involve any active collection of human data.

## Performance Evaluation

In this section, we analyze the ${\rm{\Pr }}(C({z}^{\ast }))={\rm{\Pr }}({z}^{\ast })$ success probabilities of finding the global optimal output z*, and global optimal estimate *C*(z*) in function of *R*, for an arbitrary objective function of the quantum computer with a *QG* quantum circuit, and arbitrary objective function *C*. First the $U(\overrightarrow{\theta })={U}_{B}U(\overrightarrow{\theta ^{\prime} })$ setting is discussed, then the $U(\overrightarrow{\theta })={U}_{B}$ situation is proposed.

**Figure 2:**
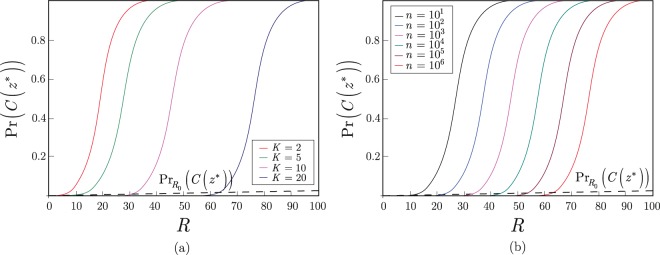
(**a**) The Pr(*C*(z*)) success probabilities for $U(\overrightarrow{\theta })={U}_{B}U(\overrightarrow{\theta ^{\prime} })$, in function of the dense measurement rounds *R*, $R=\alpha {Z}^{2}K\,{\mathrm{log}}^{4}(n)$, at $n=1000$, and $K=2,5,10,20$, ${\alpha }_{K=2}=2.47\times {10}^{-4}$, ${\alpha }_{K=5}=1.36\times {10}^{-4}$, ${\alpha }_{K=10}=8.46\times {10}^{-5}$, ${\alpha }_{K=20}=6.17\times {10}^{-5}$, and $Z=\sqrt{n}$. (**b**) The Pr(*C*(z*)) success probabilities for $U(\overrightarrow{\theta })={U}_{B}U(\overrightarrow{\theta ^{\prime} })$, in function of *R* at $K=10$, for *n* = 10^1^, 10^2^, 10^3^, 10^4^, 10^5^, 10^6^.

In Fig. 2, a dense measurement at the $U(\overrightarrow{\theta })={U}_{B}U(\overrightarrow{\theta ^{\prime} })$ case is depicted. In this case, *R* is evaluated as given in (99). In Fig. 2(a) the length of the measured quantum system is fixed to $n=1000$, while *K* varies between 5 and 20. In Fig. 2(b), the value of *K* is fixed to $K=10$, while *n* varies between $n={10}^{1}$ and $n={10}^{6}$. For a comparison the results of *R*_0_ standard measurements^13^ are also depicted in both figures with dashed gray lines (${R}_{0}=100$, ${{\rm{\Pr }}}_{{R}_{0}}(C({z}^{\ast }))={{\rm{\Pr }}}_{{R}_{0}}({z}^{\ast })\ge 0.01$).

**Figure 3:**
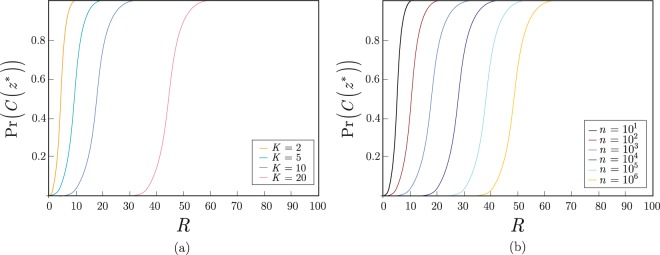
(**a**) The Pr(*C*(z*)) success probabilities for computational basis quantum states, $U(\overrightarrow{\theta })={U}_{B}$, in function of the measurement rounds *R*, $R=\gamma K\,\mathrm{log}(\frac{10n}{K})$, at $n=1000$, $K=2,5,10,20$ and $\gamma =1$. (**b**) The Pr(*C*(z*)) success probabilities for computational basis quantum states, $U(\overrightarrow{\theta })={U}_{B}$, in function of *R* at $K=10$, for *n* = 10^1^, 10^2^, 10^3^, 10^4^, 10^5^, 10^6^.

In Fig. 3, a $U(\overrightarrow{\theta })={U}_{B}$ situation is depicted. In this case, a computational basis quantum state is outputted by the *QG* structure, and *R* is evaluated as given in (124). In Fig. 3(a) the length of the measured quantum system is fixed to $n=1000$, while *K* varies between 5 and 20. In Fig. 3(b), the value of *K* is fixed to $K=10$, while *n* varies between $n={10}^{1}$ and *n* = 10^6^.

## Conclusions

Here, we defined a novel measurement technique called dense measurement for quantum computation. Dense measurement utilizes a random measurement strategy and a post-processing unit to eliminate the main drawbacks of standard measurement techniques. The dense measurement method provides two fundamental results. First, it significantly increases the success probability of finding a global optimal measurement result. Second, it radically reduces the number of measurement rounds required to determine a global optimal measurement result. We demonstrated the results through an application of dense measurements with quantum circuits that realize arbitrary unitary operations. We proved the results of dense measurement theory for the measurement of arbitrary quantum states and for the measurement of computational basis quantum states in gate-model quantum computer environment.

## Supplementary information

Supplemental Information

## Data Availability

This work does not have any experimental data.
