# Supplementary material for: Dense Quantum Measurement Theory
Source: Sci Rep. 2019 May 1;9:6755. doi: 10.1038/s41598-019-43250-2 (PMC6494868; doi:10.1038/s41598-019-43250-2)
Supplement: Supplementary file 1 — Supplemental Information [file 41598_2019_43250_MOESM1_ESM.pdf]

Dense Quantum Measurement Theory

Laszlo Gyongyosi[1,2,3] and Sandor Imre[2]

[1] School of Electronics and Computer Science
University of Southampton
Southampton SO17 1BJ, UK
[2] Department of Networked Systems and Services
Budapest University of Technology and Economics
2 Magyar tudosok krt., Budapest, H-1117 Hungary
[3] MTA-BME Information Systems Research Group
Hungarian Academy of Sciences
7 Nador st., Budapest, H-1051 Hungary

Email: l.gyongyosi@soton.ac.uk

Abstract

Quantum measurement is a fundamental cornerstone of experimental quantum computations. The main issues in current quantum measurement strategies are the high number of measurement rounds to determine a global optimal measurement output and the low success probability of finding a global optimal measurement output. Each measurement round requires preparing the quantum system and applying quantum operations and measurements with high-precision control in the physical layer. These issues result in extremely high-cost measurements with a low probability of success at the end of the measurement rounds. Here, we define a novel measurement for quantum computations called dense quantum measurement. The dense measurement strategy aims at fixing the main drawbacks of standard quantum measurements by achieving a significant reduction in the number of necessary measurement rounds and by radically improving the success probabilities of finding global optimal outputs. We provide application scenarios for quantum circuits with arbitrary unitary sequences, and prove that dense measurement theory provides an experimentally implementable solution for gate-model quantum computer architectures.

A Appendix

A.1 Abbreviations

NISQ Noisy Intermediate-Scale Quantum

QADI Quantum Adiabatic Algorithm

QAOA Quantum Approximate Optimization Algorithm

QG Quantum Gate structure of a quantum circuit

POVM Positive-Operator Valued Measure

A.2 Notations

The notations of the manuscript are summarized in Table A.1.

Table A.1: Summary of notations.

Notation	*Description*
QG	Quantum gate structure of a quantum circuit.
M	Standard measurement operator.
M_r	Measurement operator in the dense measurement procedure.
d	Dimension of the quantum system.
R_0	Measurement rounds at a standard measurement M.
R	Measurement rounds of dense measurements, $M_r = (b_1 M_B, \ldots, b_n M_B)^T$, where b_i is a random variable, $b_i \in \{0, 1\}$, $\Pr(0) = \Pr(1) = 0.5$, associated with the measurement of the i-th quantum state of the output quantum system, while M_B is a quantum measurement in the computational basis B, $b_i M_B = 0$ if $b_i = 0$, and $b_i M_B = M_B$ if $b_i = 1$.
z	Measurement output.
z^*	Global optimal measurement output.
L	Number of unitary gates in the QG quantum circuit.
$U_i(\theta_i)$	An i-th unitary gate, $U_i(\theta_i) = \exp(-i\theta_i P)$, where P is a generalized Pauli operator formulated by a tensor product of Pauli operators $\{\sigma_X, \sigma_Y, \sigma_Z\}$, while θ_i is referred to as the gate parameter associated to $U_i(\theta_i)$.
$U(\vec{\theta})$	A unitary operation realized via the quantum circuit, $U(\vec{\theta}) = U_L(\theta_L) U_{L-1}(\theta_{L-1}) \ldots U_1(\theta_1)$, where $U_i(\theta_i)$ identifies an i-th unitary gate.

$\vec{\theta}$	Gate parameter vector, a collection of gate parameters of the L unitaries, $\vec{\theta} = (\theta_1, \ldots, \theta_{L-1}, \theta_L)^T$.
C	Classical objective function of a computational problem fed into the quantum computer.
P	Generalized Pauli operator formulated by the tensor product of Pauli operators $\{\sigma_X, \sigma_Y, \sigma_Z\}$.
$\|X\rangle$	An n-length input quantum system.
X	Classical representation of $\|X\rangle$.
$\|Y\rangle$	An n-length output quantum system, $\|Y\rangle = U(\vec{\theta})\|X\rangle$.
Y	An n-dimensional output vector.
L_0	L_0-norm, the number of nonzero elements of x, $L_0(x) = \sum_{i=1}^{n} 1_{x_i \neq 0}$, or $L_0(x) = \lim\limits_{p \to 0} \sqrt[p]{\sum_{i=1}^{n} x_i^p}$.
L_1	L_1-norm, vector norm, $L_1(x) = \sum_{i=1}^{n} \|x_i\|$.
L_2	L_2-norm, Euclidean norm, $L_2(x) = \sqrt{\sum_{i=1}^{n} \|x_i\|^2}$.
ℓ^2	The ℓ^2-norm of a quantum system, $\ell^2(\|\psi\rangle) = \sqrt{\sum_x \|\psi(x)\|^2} = 1$, where $\|\psi(x)\|^2 = \Pr(x)$, and $\sqrt{\int \Pr(x)\, dx} = \sqrt{\int \|\psi(x)\|^2\, dx} = 1$.
$U(\vec{\theta'})$	An actual setting of the unitaries of QG at a particular computational basis B, to provide output $\|G\rangle = U(\vec{\theta'})\|S\rangle$, such that $U(\vec{\theta'})\|S\rangle = U(\vec{\theta})\|X\rangle$.
$\vec{\theta'}$	L-dimensional vector of the gate parameters of $U(\vec{\theta'})$.
B	Computational basis, selected such that $L_0(S) = K$, $K \ll n$, holds for the L_0-norm of S, where S is a classical representation of $\|S\rangle$.
U_B	Unitary that sets computational basis B as $U_B\|X\rangle = \|S\rangle$.
$\mathcal{P}$	Post processing unit to perform an L_1-minimization, and post-processing calculations.
$C(z^*)$	Optimal estimate of a particular objective function C fed into the quantum circuit, $C(z^*) = \max\limits_{\forall m} C(z_m)$, where $C(z_m)$ is the estimate yielded in an m-th measurement round, $m = 1, \ldots, R_0$, while z_m is the output string yielded in the m-th round.
$\Pr_{R_0}(z^*)$	Probability of finding the global optimal output z^* via R_0 standard measurement rounds.
$\Pr_R(z^*)$	Probability of finding the global optimal z^* via R dense measurement rounds.

$\Pr_{R_0} C(z^*)$	Probability of finding the global optimal $C(z^*)$ via R_0 standard measurement rounds.
$\Pr_R C(z^*)$	Probability of finding the global optimal $C(z^*)$ via R dense measurement rounds.
K	Constant, $L_0(S) \leq K$, $K \ll n$.
ε	Probability, $\varepsilon = \Pr(\delta_K \geq \chi)$, where δ_K is a constant.
$M_r^{(m)}$	Measurement operator of the m-th, $m = 1, \ldots, R$, dense measurement round $M_r^{(m)} = \left(b_1^{(m)} M_B, \ldots, b_n^{(m)} M_B\right)^T$.
$\mathcal{M}$	Measurement matrix formulated via R dense measurement rounds, $\mathcal{M} = \left(M_r^{(1)}, \ldots, M_r^{(R)}\right)$.
$\mathcal{Q}$	Matrix, $\mathcal{Q} = \mathcal{M} U(\vec{\theta'})$.
$\lvert S \rangle$	Computational basis quantum state, $\lvert S \rangle = U_B \lvert X \rangle$.
$\lvert G \rangle$	An output quantum system, $\lvert G \rangle = U(\vec{\theta'}) \lvert S \rangle$.
b_i	A random variable, $b_i = \begin{cases} 0, \text{ with } \Pr(0) = 0.5 \\ 1, \text{ with } \Pr(1) = 0.5 \end{cases}$, associated with the measurement of the i-th quantum system.
Y	An n-bit length output vector.
M_r'	A measurement operator, $M_r' = M_r U(\vec{\theta'})$.
β_C	An n-length vector, $\beta_C = (b_1, \ldots, b_n)^T$.
β_C'	An n-length vector, $\beta_C' = \beta_C U(\vec{\theta'})$.
Λ	A parameter, $\Lambda = U(\vec{\theta'}) S$.
$\tilde{S}$	Recovered computational basis vector S from $Y = \beta_C' S$ via $\mathcal{P}$.
$\tilde{\Lambda}$	An optimal value of Λ evaluated from $\tilde{S}$, $\tilde{\Lambda} = U(\vec{\theta'}) \tilde{S}$.
Y^R	Measurement output matrix of R measurement rounds, $Y^R = \mathcal{Q} \lvert S \rangle = \left(Y^{(1)}, \ldots, Y^{(R)}\right)$, where $Y^{(m)}$ is the measurement result vector of the m-th round.
ξ	Error probability of finding z^* at the end of the R rounds, $\Pr(z \neq z^*) = \xi$.
C_1, C_2	Sub-Gaussian parameters, $C_1, C_2 > 0$.
δ_K	K-th restricted isometry constant.

$[n]$	Set of natural numbers not exceeding n, $[n] = \{1, \ldots, n\}$.
Υ	A subset.
H	Hermitian matrix.
Ω, κ, χ	Parameters of the dense measurement procedure.
V, W, D	Parameters of the dense measurement procedure.
$\mathcal{B}_\Upsilon$	Unit ball.
Γ	A finite subset of $\mathcal{B}_\Upsilon$.
$\delta_K < \chi$	Event associated with probability $\Pr(\delta_K < \chi) = 1 - \varepsilon$, where δ_K is the K-th restricted isometry constant.
$U(\vec{\theta}_{q,k})$	A q-th element of the k-th column of $U(\vec{\theta})$.
Z	Constant, $Z \geq \sqrt{n} \max\limits_{k,q \in [n]} \left\| U(\vec{\theta}_{q,k}) \right\|$.
α	Constant, $\alpha > 0$.
u_k	A k-th column of $U(\vec{\theta})$, $k = 1, \ldots, n$.
v_k	A normalized k-th column of $U(\vec{\theta})$, $v_k = \sqrt{n} u_k$, $k = 1, \ldots, n$.
φ_{kl}	Inner product of two normalized columns v_k and v_l, as $\varphi_{kl} = \left\langle \frac{1}{\sqrt{n}} v_k, \frac{1}{\sqrt{n}} v_l \right\rangle = \langle u_k, u_l \rangle$.
$u_{i,j}$	A unitary $u_{i,j} = U(\vec{\theta}_{j,i})$, j-th element of the i-th column of $U(\vec{\theta})$.
$v_{i,j}$	A normalization of $u_{i,j}$, $v_{i,j} = \sqrt{n} U(\vec{\theta}_{j,i})$.
u'_k	A k-th column of $U(\vec{\theta}')$.
b_q	A q-th column of U_B.
$\mathcal{P}_{Q_R}$	Projector, selects a subset of $U(\vec{\theta})$ in the R rounds, where $Q_R \subset [n]$ is a subset of R elements selected uniform at random from all subsets of $[n]$ of cardinality R, $\|Q_R\| = R$.
ξ^*	Error probability associated with the selection of rows uniformly and independently at random from $U(\vec{\theta})$.
Q_R	A subset of R elements selected uniform at random from all subsets of $[n]$ of cardinality R, $Q_R \subset [n]$, $\|Q_R\| = R$.
Q'_R	A subset of R elements, elements are selected independently and uniformly at random from $[n]$, $Q'_R \subset [n]$, $\|Q'_R\| = R$.
Q_k	A subset of $k \leq R$ selected uniform at random from all subsets of $[n]$ of cardinality k, $Q_k \subset [n]$, $\|Q_k\| = k$.

$\mathcal{E}(Q)$	Event that the L_1-minimization in $\mathcal{P}$ fails.
$\mathcal{D}(\cdot)$	A distribution.
Λ^*	An optimal $\tilde{\Lambda}$ determined via $\mathcal{P}$.
δ_{2K}	$2K$-th restricted isometry constant.
